# Point Shear Wave Elastography for Assessment of Liver Stiffness in Normal Individuals and in Patients With Non-alcoholic Fatty Liver Disease

**DOI:** 10.7759/cureus.70711

**Published:** 2024-10-02

**Authors:** Parag V Patil, Sravya Julakanti, Rajshree U Dhadve

**Affiliations:** 1 Radiodiagnosis, Dr. D. Y. Patil Medical College, Hospital and Research Centre, Pune, IND

**Keywords:** fatty liver, liver stiffness, non‐alcoholic fatty liver disease, point shear wave elastography, ultrasound elastography

## Abstract

Background

Non-alcoholic fatty liver disease (NAFLD) is an increasing health issue worldwide, driven by rising rates of diabetes, obesity, and hypertriglyceridemia. Often asymptomatic, NAFLD is diagnosed through blood tests, imaging, and sometimes liver biopsy. Some cases advance to non-alcoholic steatohepatitis (NASH), which can lead to complications like liver cirrhosis and liver failure. While liver biopsy is the standard test for diagnosis, non-invasive methods such as shear wave elastography (SWE) offer a simpler and more reproducible alternative for diagnosing NAFLD. This is crucial for early intervention and preventing the progression of liver damage.

Objectives

The objectives of the study were to measure and compare liver stiffness in healthy individuals and patients with NAFLD using point shear wave elastography (pSWE), as well as to correlate liver stiffness in NAFLD patients with the ultrasonographic grades of fatty liver.

Materials and methods

This observational study was carried out at Dr. D. Y. Patil Medical College, Hospital and Research Centre in Pune, India, from December 2022 to April 2024. The study involved 82 participants in total, with 41 patients diagnosed with NAFLD (cases) and 41 healthy individuals with a sonographically normal liver (controls). pSWE was performed on each participant to measure liver stiffness, with results expressed in kilopascals (kPa). The procedure was conducted using a Samsung HS70A ultrasound machine (Samsung Electronics Pvt. Ltd., Seoul, South Korea). Data analysis was performed using IBM SPSS Statistics for Windows, Version 26.0 (Released 2019; IBM Corp., Armonk, New York, USA). Non-parametric tests, such as the Mann-Whitney test and Kruskal-Wallis test, were used to evaluate the significance of differences. A p-value of less than 0.05 was considered statistically significant.

Results

The mean liver stiffness, measured in kilopascals (kPa), was higher in NAFLD patients (cases) (10±5.1 kPa) than in normal individuals (controls) (4.4±0.7 kPa). This difference was statistically significant, with a p-value of less than 0.001. A positive correlation (rho=0.848, p<0.001) was found between the ultrasonographic grade of fatty liver and liver stiffness in NAFLD patients.

Conclusion

Our study demonstrated that individuals with NAFLD exhibited significantly higher liver stiffness compared to healthy individuals, as measured by ultrasound SWE. These findings suggest that pSWE could serve as a valuable, non-invasive diagnostic tool for assessing liver stiffness in NAFLD patients. Additionally, pSWE holds the potential for evaluating and monitoring the progression of the disease. However, further research with larger sample sizes is necessary to determine the prognostic significance of liver stiffness in these patients.

## Introduction

Non-alcoholic fatty liver disease (NAFLD) is marked by fat accumulation in over 5% of liver cells and is linked to metabolic conditions like obesity and type II diabetes mellitus. It occurs without the presence of other chronic liver diseases or significant alcohol consumption [1]. The incidence of NAFLD is rising quickly, especially in developed countries. Several factors contribute to this increase, including increasing obesity rates, a surge in obesity among the pediatric population, sedentary lifestyles, the widespread use of unhealthy fast food, and longer life expectancies [2].

With a worldwide incidence of around 25% of adults, NAFLD has become the principal liver ailment over the past four decades [3,4]. Due to its widespread prevalence worldwide, NAFLD has become the rapidly increasing cause of liver-related mortality [5]. Liver biopsies are considered the definitive method for diagnosing NAFLD. However, the invasiveness and cost of this procedure restrict its use [6]. These limitations have highlighted the need for non-invasive imaging tests to diagnose and grade NAFLD. Non-invasive options like serum biomarkers, ultrasonography, ultrasound elastography, magnetic resonance elastography, and magnetic resonance-based fat quantification were used as alternatives.

Conventional ultrasound is used for the initial screening of fatty liver. Recently, ultrasound elastography has garnered attention as an innovative and non-intrusive technique for evaluating liver stiffness and fibrosis. This advanced technique offers the potential to replace liver biopsy, providing a safer and less invasive option for evaluating liver stiffness [7]. Ultrasound elastography is a non-invasive imaging test designed to assess the mechanical properties of soft tissues, specifically their stiffness or elasticity. By applying a mechanical force and measuring the resulting displacements, this technique can infer tissue elasticity, offering valuable insights into various medical conditions [8]. Its usefulness originates from the observation that tissues with disease are more rigid and less elastic than the adjoining normal tissues. Furthermore, it is well known that elasticity changes more quickly in pathologically changed tissues than it does in other parameters [9].

Ultrasound elastography is broadly classified into two main categories: strain imaging and shear wave imaging. Strain imaging provides a qualitative assessment of tissue elasticity by measuring deformation under mechanical stress, while shear wave imaging offers a quantitative evaluation by analyzing the propagation velocity of shear waves generated within the tissue. The main types of shear wave elastography (SWE) are transient elastography (TE), point shear wave elastography (pSWE), and two-dimensional shear wave elastography (2D-SWE). These methods are utilized in diverse clinical applications, such as assessing liver fibrosis, characterizing breast and thyroid lesions, and evaluating musculoskeletal disorders [10,11]. First created in 2005, SWE is incorporated into ultrasound machines to provide simultaneous liver inspection, morphological ultrasound, and quantitative elastography evaluation of liver fibrosis [12]. Elastography operates on the principle that certain conditions make the tissues "stiffer" than normal tissues; therefore, an increase in shear wave velocity indicates greater tissue stiffness [13].

The study aimed to assess and compare liver stiffness in healthy individuals and patients with NAFLD. Additionally, the study also aimed to assess the association between liver stiffness measurements and the ultrasonographic grades of fatty liver in patients with NAFLD.

## Materials and methods

This observational study was conducted at Dr. D. Y. Patil Medical College, Hospital and Research Centre, Pune, India from December 2022 to April 2024. The study included a total of 82 participants. The study was granted ethical clearance from the Institutional Ethics Committee of Dr. D. Y. Patil Medical College, Hospital and Research Centre with the reference number- IESC/PGS/2022/172. Informed and written consent was obtained from all the research subjects.

Inclusion and exclusion criteria

Cases included patients who exhibited sonographically raised liver echotexture and had no history of alcohol intake. Controls were healthy individuals with sonographically normal liver echotexture and no history of alcohol intake. Participants under the age of 18, those with alcoholic liver disease, or those unable to hold their breath were excluded. Additionally, individuals having metabolic or systemic diseases that could affect the liver were excluded from the control group.

Technique

**Figure 1:**
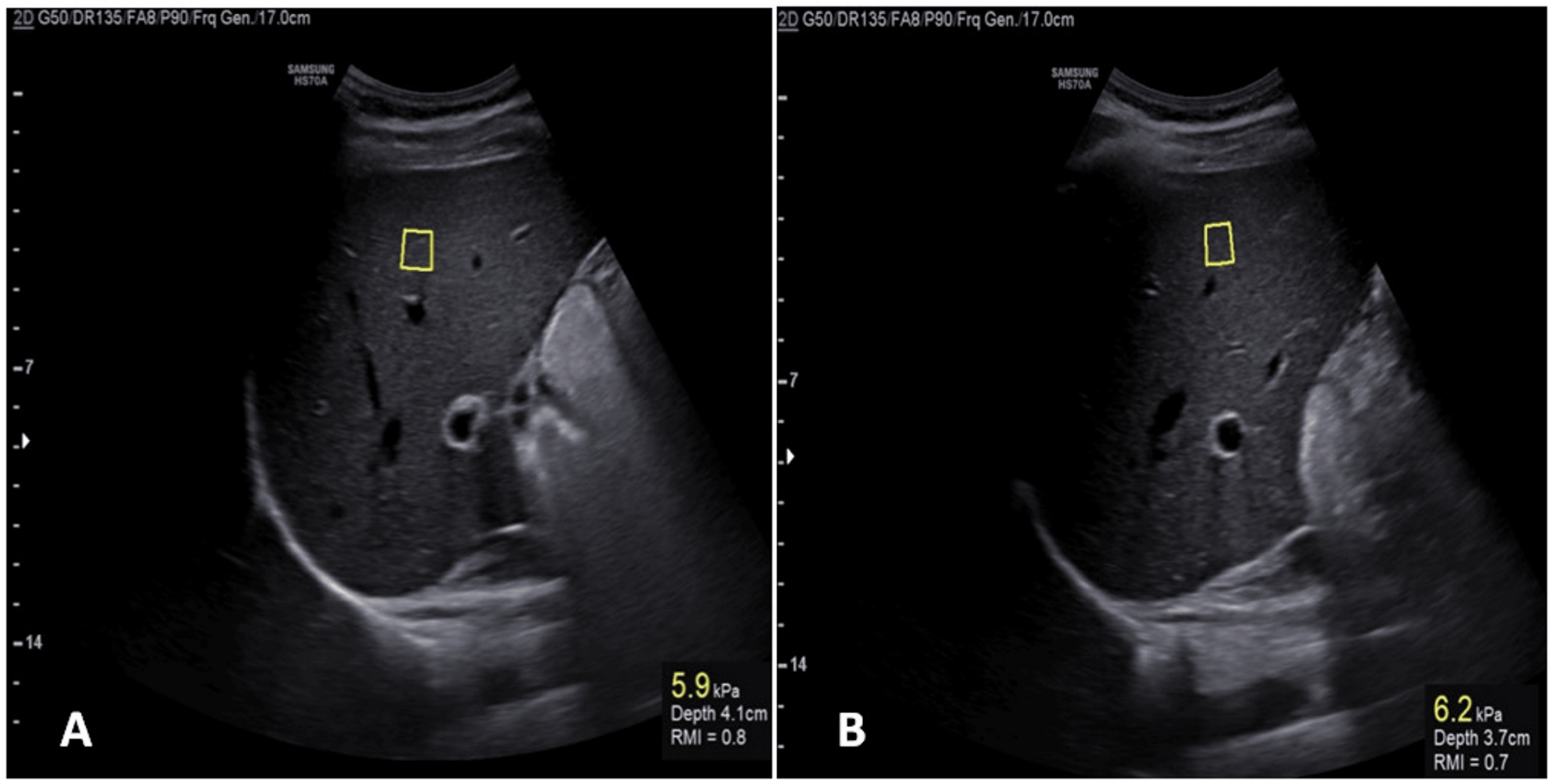
Ultrasound image utilized to assess liver stiffness. The ROI is marked by a small square (yellow box), and the liver stiffness is measured within this area ROI: region of interest; pSWE: point shear wave elastography

The ultrasound examinations were performed using a Samsung HS70A ultrasound machine (Samsung Electronics Pvt. Ltd., Seoul, South Korea). The patients were positioned supine with their right arm fully abducted to broaden the space between the ribs, facilitating optimal access for the ultrasound probe, which was placed in the ideal intercostal space to visualize the liver parenchyma. An ultrasound examination using brightness-mode (B-mode) was conducted to assess and grade fatty liver, incorporating grades from mild to severe, based on the observed hepatic echogenicity and attenuation. A curved array transducer was used for the pSWE, and the region of interest (ROI) was positioned on the right lateral side, specifically the right lobe of the liver, at a depth of less than 6 cm while avoiding vessels and maintaining a 2 cm distance from the liver capsule. Measurements were taken during suspended respiration, with 10 readings obtained in the ROI. The average median value was then recorded as the final liver stiffness measurement, expressed in kilopascals (kPa). Figure 1 shows an ultrasound image utilizing pSWE to assess liver stiffness. 

Statistical analysis

Data analysis was performed using IBM SPSS Statistics for Windows, Version 26.0 (Released 2019; IBM Corp., Armonk, New York, United States). The categorical data were reported in the form of frequency and percentage. For continuous data, the mean (SD) and median (IQR) were computed. Non-parametric tests were used because the continuous variables were not normally distributed. The Mann-Whitney U test was used to determine the statistical significance of mean differences between cases and controls. The Kruskal-Wallis test was utilized to examine the significance of variations in liver stiffness across various BMI categories and fatty liver grades. A p-value less than 0.05 was deemed statistically significant.

## Results

Study population

In all, 82 individuals were involved, comprising 41 cases (NAFLD patients) and 41 controls (healthy individuals). Each group represented 50% of the total study population.

In the NAFLD cases group, participants’ ages varied from 26 to 73 years, with a mean age of 46.4 years (±11.4 years). In the control group, ages varied from 23 to 76 years, with a mean age of 44 years (±12.8 years). The median age for the case group was 47 years and 42 years for the control group (Table 1).

**Table 1:** The age distribution of cases and controls

Statistics	Cases	Controls
Mean (years)	46.4	44.0
Median (years)	47.0	42.0
Std. Deviation (years)	11.4	12.8

In the cases group, 19 (46.3 %) individuals were male and 22 (53.7%) individuals were female. In the control group, 18 (43.9%) individuals were male and 23 (56.1%) individuals were female. The total number of males in the study population was 37 (45.1%), and the total number of females was 45 (54.9%). The majority were female in both the cases (53.7%) as well as the control groups (56.1%) (Table 2).

**Table 2:** The gender distribution of the study population

Gender	Groups	N (%)
Males	Cases	19 (46.3%)
	Controls	18 (43.9%)
	Total	37 (45.1%)
Females	Cases	22 (53.7%)
	Controls	23 (56.1%)
	Total	45 (54.9%)

Clinical characteristics

Among the 41 cases, 18 individuals (43.9%) had diabetes mellitus, 7 individuals (17.1%) had hypertension, 8 individuals (19.5%) had dyslipidemia, 25 individuals (61%) had abnormal liver function tests (LFTs), and 23 individuals (56.1%) had an abnormal lipid profile. None of the controls had laboratory abnormalities (Table 3).

**Table 3:** The distribution of clinical and laboratory abnormalities among cases LFT: liver function tests

Clinical factors	N (%)
Diabetes mellitus	18 (43.9%)
Hypertension	7 (17.1%)
Dyslipidemia	8 (19.5%)
Abnormal LFT	25 (61%)
Abnormal Lipid Profile	23 (56.1%)

The grading of body mass index (BMI) used in the study was as follows: normal (18.5-24.9), overweight (25-29.9), and obese (30 and above). In the cases, 41.5 % had a normal BMI, and 39% and 19.5% were overweight and obese, respectively. In contrast, all the controls had normal BMI (Table 4).

**Table 4:** The grading of BMI among cases and controls BMI: body mass index

BMI	Groups	N (%)
Normal	Cases	17 (41.5%)
	Controls	41 (100%)
	Total	58 (70.7%)
Overweight	Cases	16 (39.0%)
	Controls	0 (0%)
	Total	16 (19.5%)
Obese	Cases	8 (19.5%)
	Controls	0 (0%)
	Total	8 (9.8%)

Grades of fatty liver on brightness-mode ultrasound

In the cases group, ultrasonography revealed Grade 1 fatty liver in 25 individuals (61%), Grade 2 fatty liver in 13 individuals (31.7%), and Grade 3 fatty liver in 3 individuals (7.3%). None of the controls showed fatty liver (Table 5).

**Table 5:** The distribution of grades of fatty liver on ultrasonography among the cases and controls

Fatty liver grade	Groups	N (%)
Normal	Cases	0 (0%)
	Controls	41 (100%)
	Total	41 (50%)
Grade 1	Cases	25 (61%)
	Controls	0 (0%)
	Total	25 (30.5%)
Grade 2	Cases	13 (31.7%)
	Controls	0 (0%)
	Total	13 (15.9%)
Grade 3	Cases	3 (7.3%)
	Controls	0 (0%)
	Total	3 (3.7%)

Liver stiffness

The mean liver stiffness in the control group was 4.4±0.7 kPa, with the highest value at 5.6 kPa and the lowest value at 2.8 kPa. The mean liver stiffness in the NAFLD cases was 10±5.1 kPa, with the highest value at 32.5 kPa and the lowest value at 5.7 kPa. The median liver stiffness was 4.4 kPa in the controls and 8.2 kPa in the cases (Table 6). 

**Table 6:** Liver stiffness values by elastography in cases and controls

Statistics	Cases	Controls
Mean (kPa)	10	4.4
Median (kPa)	8.2	4.4
Std. deviation (kPa)	5.1	0.7

**Figure 2:**
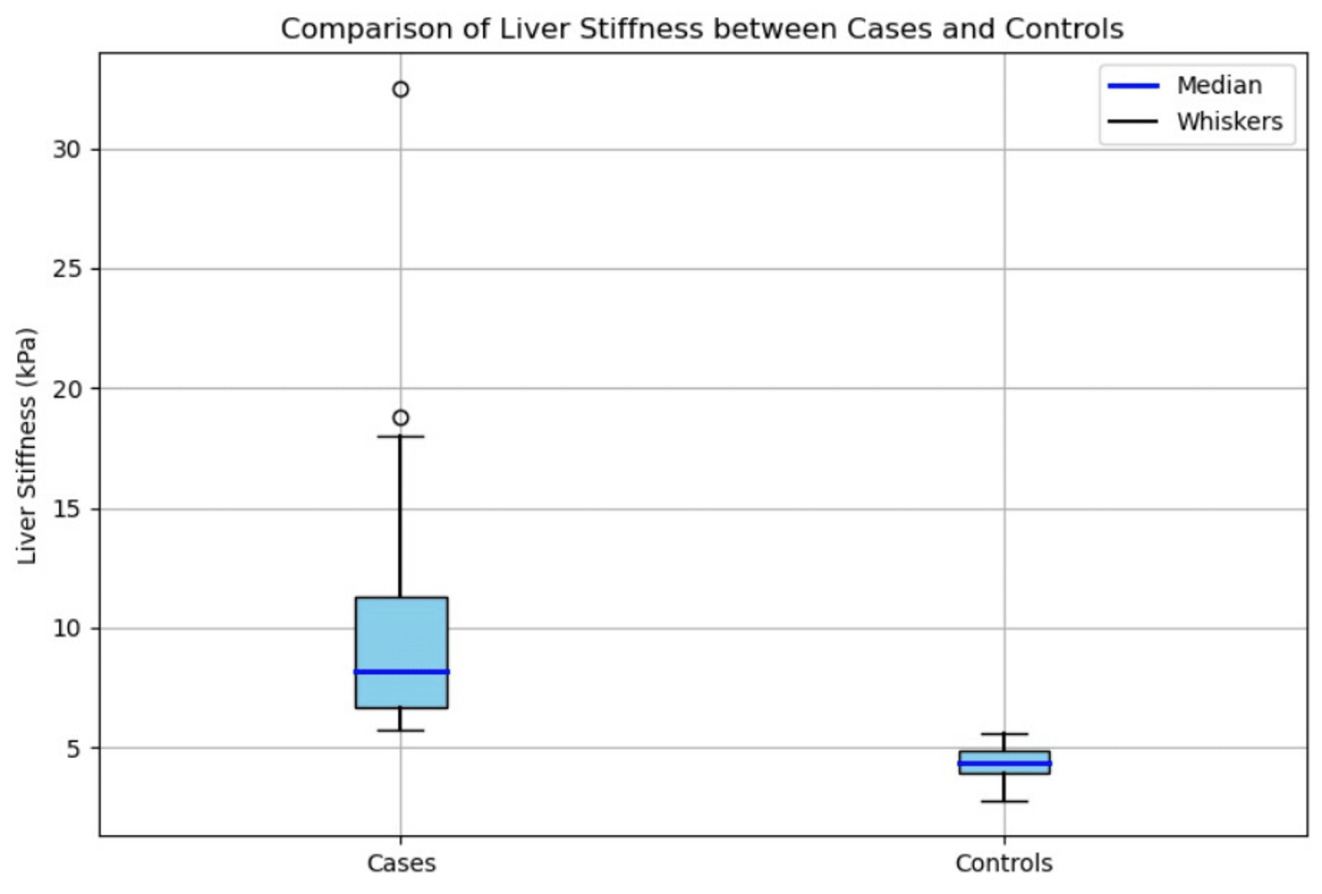
Box-and-whisker plot showing the comparison of liver stiffness between cases and controls The box-and-whisker plot demonstrates that the cases (NAFLD patients) exhibited higher median liver stiffness values as well as a broader range compared to the controls (normal individuals). The blue boxes represent the IQR of liver stiffness for each group, with the dark blue lines indicating the median values. The black whiskers extend to the minimum and maximum values within 1.5 times the IQR from the first and third quartiles. The circles outside of the whiskers represent the outliers. IQR: interquartile range; NAFLD: non-alcoholic fatty liver disease

There was a notable difference in the median liver stiffness measured in the cases and controls, with a p-value of less than 0.001. The median liver stiffness was significantly higher among the NAFLD patients than the healthy controls. Figure 2 shows a box-and-whisker plot depicting the comparison of liver stiffness between the cases and controls.

BMI and liver stiffness

The mean liver stiffness in normal-weight individuals was 5.7±2.8 kPa, in overweight individuals was 10.9±7.0 kPa, and in obese individuals was 10.2±2.9 kPa. The median liver stiffness in normal, overweight, and obese individuals was 4.9 kPa, 8.0 kPa, and 9.8 kPa, respectively. There was a notable difference in the median liver stiffness based on patients’ BMI (p<0.001). Obese and overweight patients had significantly higher median liver stiffness compared to those with normal weight (p<0.001) (Table 7).

**Table 7:** Liver stiffness values among different BMI categories

Statistics	Normal	Overweight	Obese
Mean (kPa)	5.7	10.9	10.2
Median (kPa)	4.9	8.0	9.8
Std. deviation (kPa)	2.8	7.0	2.9

Grades of fatty liver and liver stiffness

The median liver stiffness among normal individuals, Grade 1, Grade 2, and Grade 3 fatty liver patients was 4.4 kPa, 7.1 kPa, 12.4 kPa, and 18.8 kPa, respectively. There was a significant difference in the median liver stiffness associated with varying grades of fatty liver (p<0.001). Grade 1, 2, and 3 fatty liver patients had median liver stiffness higher than the normal individuals (Table 8).

**Table 8:** Liver stiffness values among different grades of fatty liver

Statistics	Normal	Grade 1 fatty liver	Grade 2 fatty liver	Grade 3 fatty liver
Mean (kPa)	4.4	7.2	12.3	23.1
Median (kPa)	4.4	7.1	12.4	18.8
Std. deviation (kPa)	0.7	1.2	2.4	8.2

**Figure 3:**
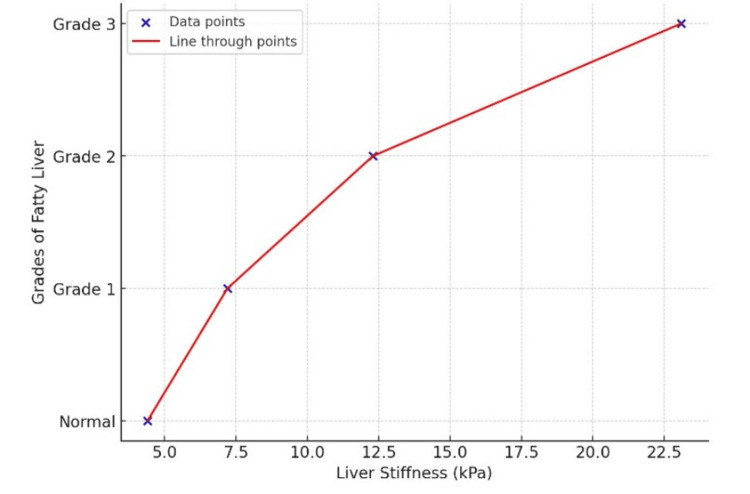
The positive statistical correlation between ultrasonographic grades of fatty liver and liver stiffness (kPa). The data points are represented by blue marks, and the red line represents the line drawn through the data points

Among NAFLD patients, a significant positive correlation existed between fatty liver grade and liver stiffness as assessed by pSWE (rho=0.848, p<0.001) (Figure 3).

## Discussion

Liver stiffness measurement is a crucial tool for quantifying hepatic fibrosis in patients diagnosed with NAFLD. Elastography techniques based on ultrasound, particularly SWE, have emerged as valuable methods for assessing liver stiffness. SWE allows for real-time evaluation by analyzing the propagation velocity of shear waves generated by ultrasound pulses, combined with anatomical B-mode ultrasound imaging. This technique provides qualitative and quantitative assessments of tissue elasticity, offering a comprehensive measure of liver fibrosis [14].

Ultrasound-based elastography techniques, TE, pSWE, and 2D-SWE, each offer unique approaches to measuring liver stiffness. TE, also known as Fibroscan, measures liver stiffness by evaluating the velocity of a shear wave produced by an acoustic pulse and is widely used to evaluate liver fibrosis and cirrhosis. The pSWE generates a localized shear wave at a specific point in the tissue, with the wave's velocity used to determine tissue elasticity and provide quantitative values of stiffness at a targeted area. The 2D-SWE maps tissue stiffness over a two-dimensional area, offering a comprehensive view of elasticity distribution within an ROI and helping to assess the variability of stiffness across different tissue areas [15].

In our study, we utilized pSWE to measure liver stiffness, observing a significant difference between NAFLD patients and healthy individuals. The mean liver stiffness was 10±5.1 kPa in NAFLD patients compared to 4.4±0.7 kPa in healthy controls (p<0.001). These findings underscore pSWE's potential as a dependable non-invasive tool method for determining liver stiffness in NAFLD patients. Our results coincide with the findings reported by Kristian Podrug et al. (2021), who discovered similar liver stiffness values in a larger cohort [16].

Further analysis of liver stiffness measurements using pSWE in NAFLD patients revealed a significant increase in stiffness values correlating with the severity of fatty liver: 7.2 kPa for Grade 1, 12.3 kPa for Grade 2, and 23.1 kPa for Grade 3. This finding underscores the effectiveness of pSWE in distinguishing between varying degrees of liver fibrosis associated with fatty liver disease. Similar findings were observed in studies by Gupta N et al. (2019) and Chaudhari et al. (2023) [17,18].

The current study had several limitations. The reliance on self-reported alcohol intake history for case definition could have introduced bias due to under-reporting or misclassification. The relatively small sample size in this single-center study restricts the ability to generalize the observations to broader populations. Additionally, variability in pSWE measurements could arise from operator technique and patient positioning, potentially affecting the consistency of the results.

A meta-analysis by Weixi Jiang et al. (2018) further supports the significant value of non-invasive techniques for staging hepatic fibrosis in NAFLD patients. This meta-analysis, which included studies on TE and pSWE, concluded that both methods are accurate and reliable for assessing liver fibrosis, particularly in diagnosing advanced fibrosis and cirrhosis. These findings underscore the important role of pSWE and TE in clinical practice, supporting their use as diagnostic tools in managing NAFLD and aiding in effective treatment planning [19].

In patients with evident hepatic cirrhosis, measuring liver stiffness offers two significant benefits. Firstly, increased stiffness can be diagnostically valuable for identifying large varices, which are critical to monitor due to their risk of bleeding [20]. Secondly, higher liver stiffness values may predict other complications and liver-related mortality [21]. Ultrasound elastography is, therefore, poised to become a primary tool for the ongoing assessment of NAFLD patients. This underscores its importance in regular monitoring and long-term management of liver conditions.

## Conclusions

Our study demonstrated that individuals with NAFLD exhibited significantly higher liver stiffness compared to healthy individuals, as measured by ultrasound SWE. These findings suggest that pSWE could serve as a valuable, non-invasive diagnostic tool for assessing liver stiffness in NAFLD patients. Additionally, pSWE holds the potential for evaluating and monitoring the progression of the disease. However, further research with larger sample sizes is necessary to determine the prognostic significance of liver stiffness in these patients.
